# Supplementary material for: Proteotoxicity caused by perturbed protein complexes underlies hybrid incompatibility in yeast
Source: Nat Commun. 2022 Jul 29;13:4394. doi: 10.1038/s41467-022-32107-4 (PMC9338014; doi:10.1038/s41467-022-32107-4)
Supplement: Supplementary file 1 — Supplementary Information [file 41467_2022_32107_MOESM1_ESM.pdf]

Supplementary Information for

Proteotoxicity caused by perturbed protein complexes underlies hybrid incompatibility in yeast

Krishna B. S. Swamy[1,2], Hsin-Yi Lee[1], Carmina Ladra[1], Chien-Fu Jeff Liu[1], Jung-Chi Chao[1], Yi-Yun Chen[3] and Jun-Yi Leu[1*]
Corresponding author email: jleu@imb.sinica.edu.tw

Supplementary Figures

Supplementary Fig. 1: The slow recovery of replacement lines from heat stress is not due to a failure to induce heat shock chaperones or cell death.
Supplementary Fig. 2: F1 hybrid diploids demonstrate proteotoxic stress.
Supplementary Fig. 3: W303-*SSD1* is not the primary cause of intrinsic proteotoxic stress in replacement lines.
Supplementary Fig. 4: The intrinsic proteotoxic stress levels in replacement lines are correlated with the number of complexes having subunits encoded on the replaced chromosomes, even when the most defective line (16L) is excluded.
Supplementary Fig. 5: Illustrative representation of elution pattern differences (EPDs) using 16L as an example.
Supplementary Fig. 6: Similar destabilized protein complexes are observed in 8+15L and 16L cells even after excluding the proteins encoded by replaced chromosomes.
Supplementary Fig. 7: Protein abundances of complexes having subunits encoded on replaced chromosomes are significantly reduced even after excluding the proteins encoded by replaced chromosomes.
Supplementary Fig. 8: Assembled RNA Polymerase III is reduced and Proteasomes exhibit normal activity but are overburdened by destabilized complex components in replacement lines.
Supplementary Fig. 9: Protein complexes create a microenvironment for epistasis between subunits.

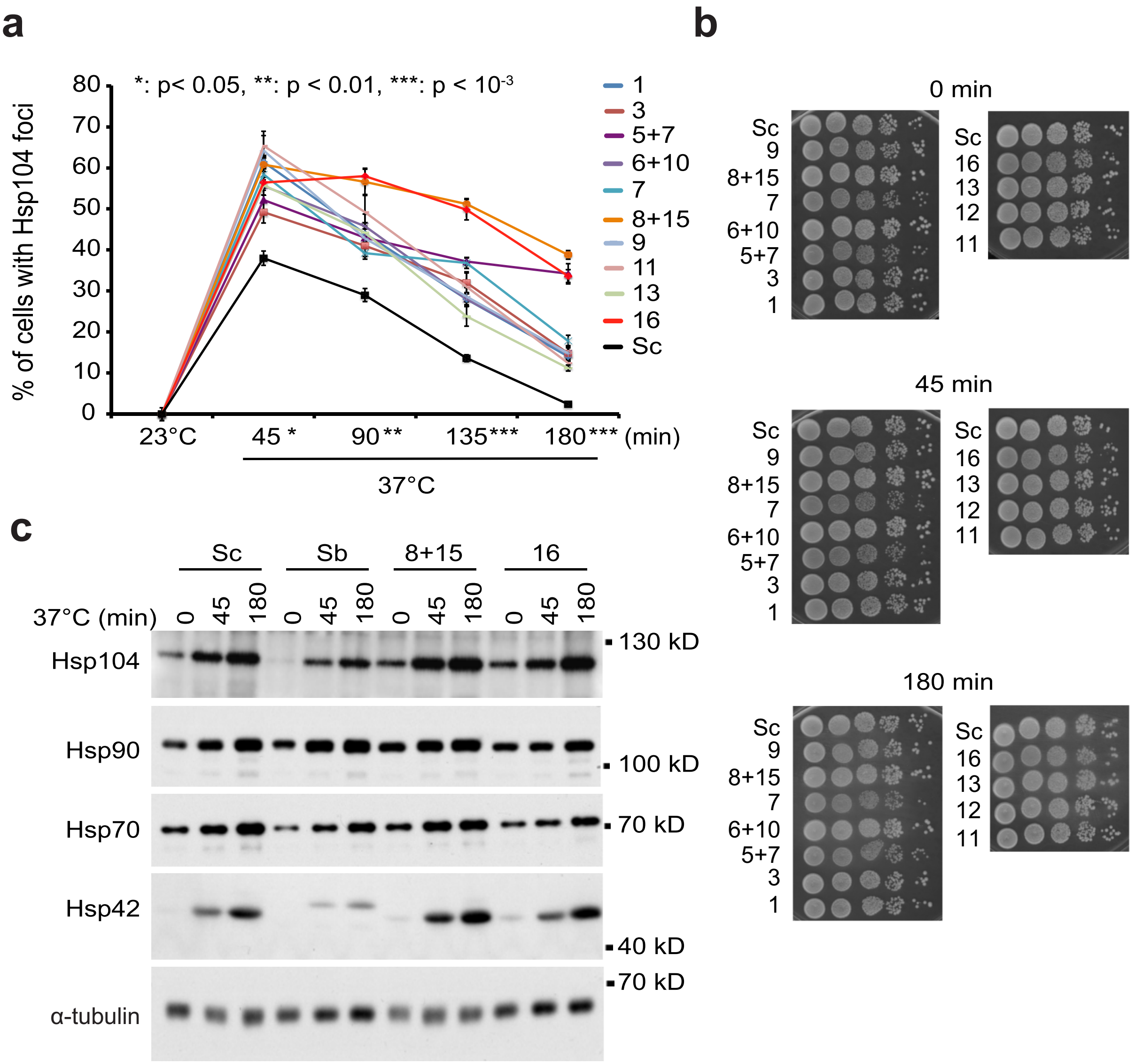

Supplementary Fig. 1: The slow recovery of replacement lines from heat stress is not due to a failure to induce heat shock chaperones or cell death.

a, A majority of the *S. cerevisiae* parent and replacement line cells harbored Hsp104-mCherry aggregates immediately upon being shifted from 23°C to 37°C (0 min to 45 min). However, whereas *S. cerevisiae* parental cells could adapt to the heat stress condition of 37°C and dissolve all aggregates within 180 min, significant proportions of replacement line cells still harbored protein aggregates after 180 min. The data are presented as mean values +/- SEM (n = 8; SEM, N ≥ 500 cells per time-point. *: p-value < 0.05, **: p-value < 0.01, ***: p-value < 10^{-3}, one-sided Student's t-test). **b,** Cell viability of replacement lines is similar to that of the *S. cerevisiae* parent line. Spot assays were performed for cells incubated at 37°C for 0, 45, and 180 min. The numbers on the left correspond to the replaced chromosomes. **c,** Western blots from parental *S. cerevisiae* (Sc) and *S. bayanus* var *uvarum* (Sb), as well as the top two aggregate-containing replacement lines 16L and 8+15L, indicate that abundances of heat shock proteins are similar across all four lines after heat treatment (37°C). **α**-tubulin acted as a sample processing control. Similar patterns were reproducible in three independent

experiments. Source data and detailed statistical information are provided as a Source Data file.

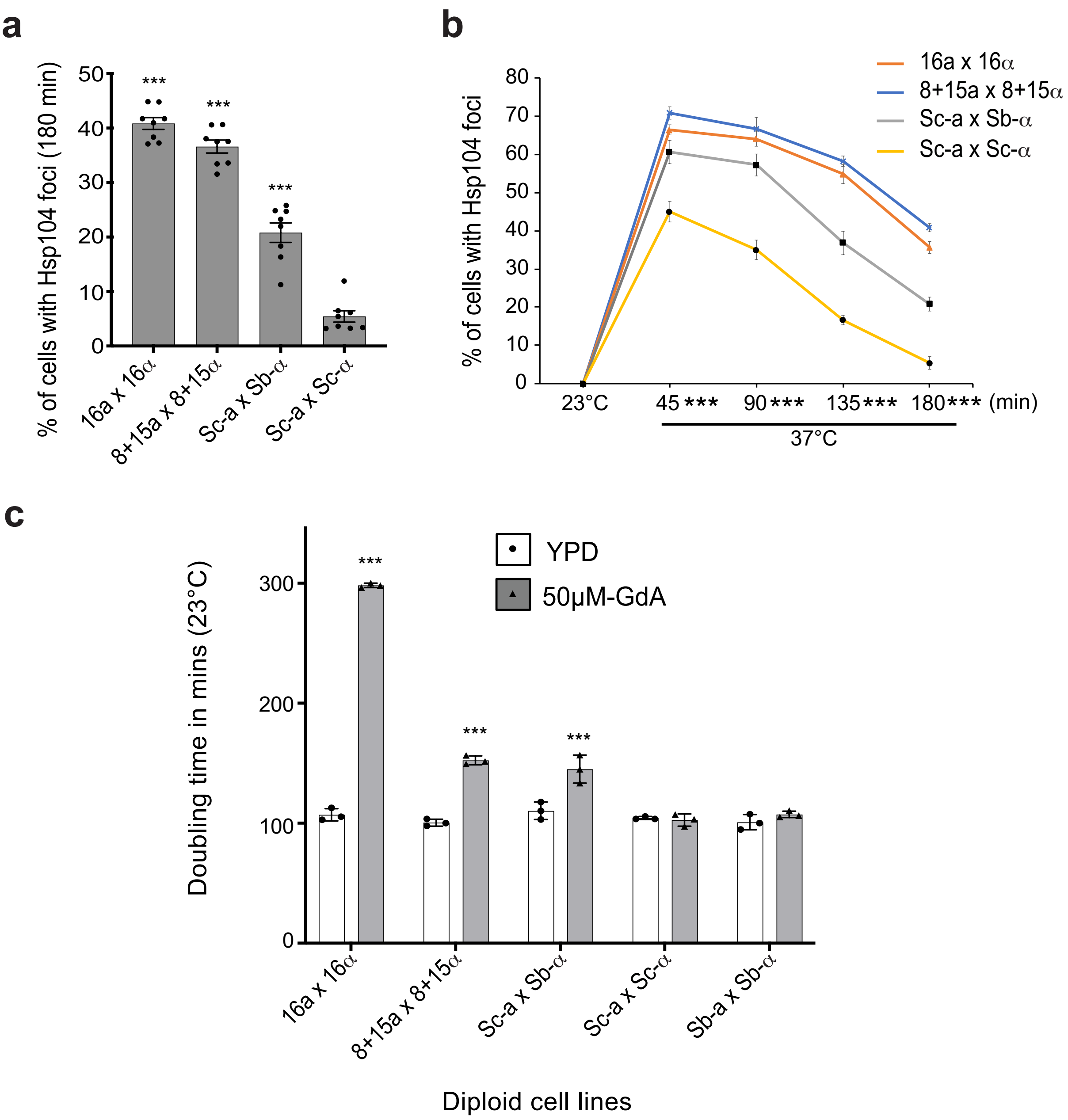

Supplementary Fig. 2: F1 hybrid diploids demonstrate proteotoxic stress.
a and **b**, F1 hybrid diploids derived from crossing haploid Sc with haploid Sb have a significantly higher percentage of cells harboring protein aggregates than diploid Sc. (n = 8; SEM, N ≥ 500 cells per time-point, one-sided Student's t-test, *: p-value < 0.05, **: p-value < 10^{-2}, ***: p-value < 10^{-3}). **c**, F1 hybrid diploids exhibit significant fitness defects compared to diploid Sc and Sb under the treatment of the Hsp90 inhibitor Geldanamycin (50 µM-GdA), at 23°C (n = 3, ***: p-value < 10^{-3}; one-sided Student's t-test between *S. cerevisiae* and F1 hybrid diploids, and replacement lines. The data are presented as mean values +/- SEM. Source data and detailed statistical information are provided as a Source Data file.

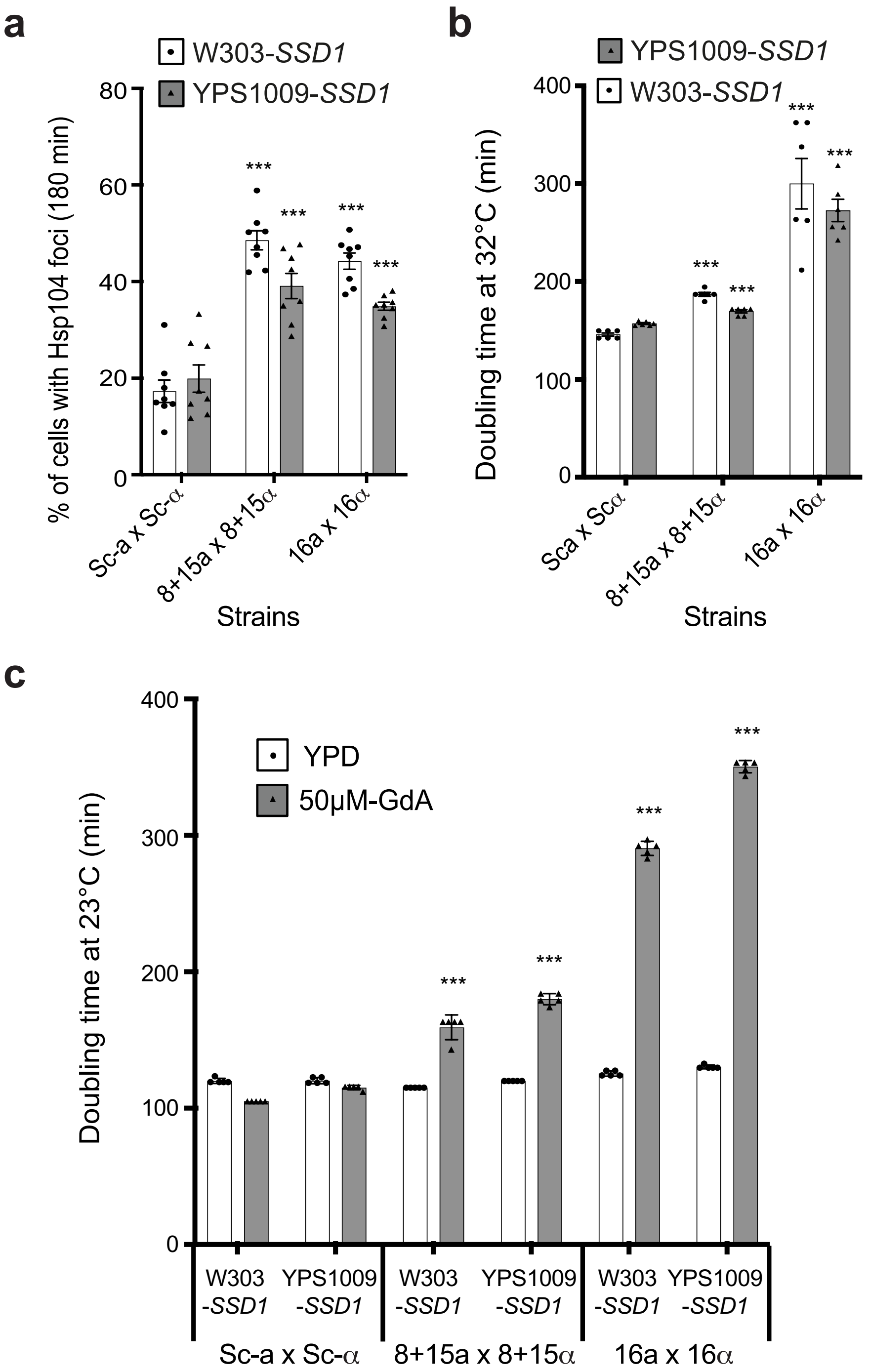

Supplementary Fig. 3: W303-*SSD1* is not the primary cause of intrinsic proteotoxic stress in replacement lines.

a, The percentage of cells containing protein aggregates in 8+15L and 16L cells is still significantly higher than that in Sc cells when cells carry $SSD1^{YPS1009}$ (n = 8). **b**, the growth defects due to mild heat stress (32°C; n = 6) or **c**, the growth defects due to a mild dose of Geldanamycin (50 μM GdA; n = 5) do not significantly change when $SSD1^{YPS1009}$ is introduced into 8+15L and 16L cells. ***: p-value < 10^{-3}; one-sided Student's t-test between diploid *S. cerevisiae* and replacement lines. The data are presented as mean values +/- SEM. Source data and detailed statistical information are provided as a Source Data file.

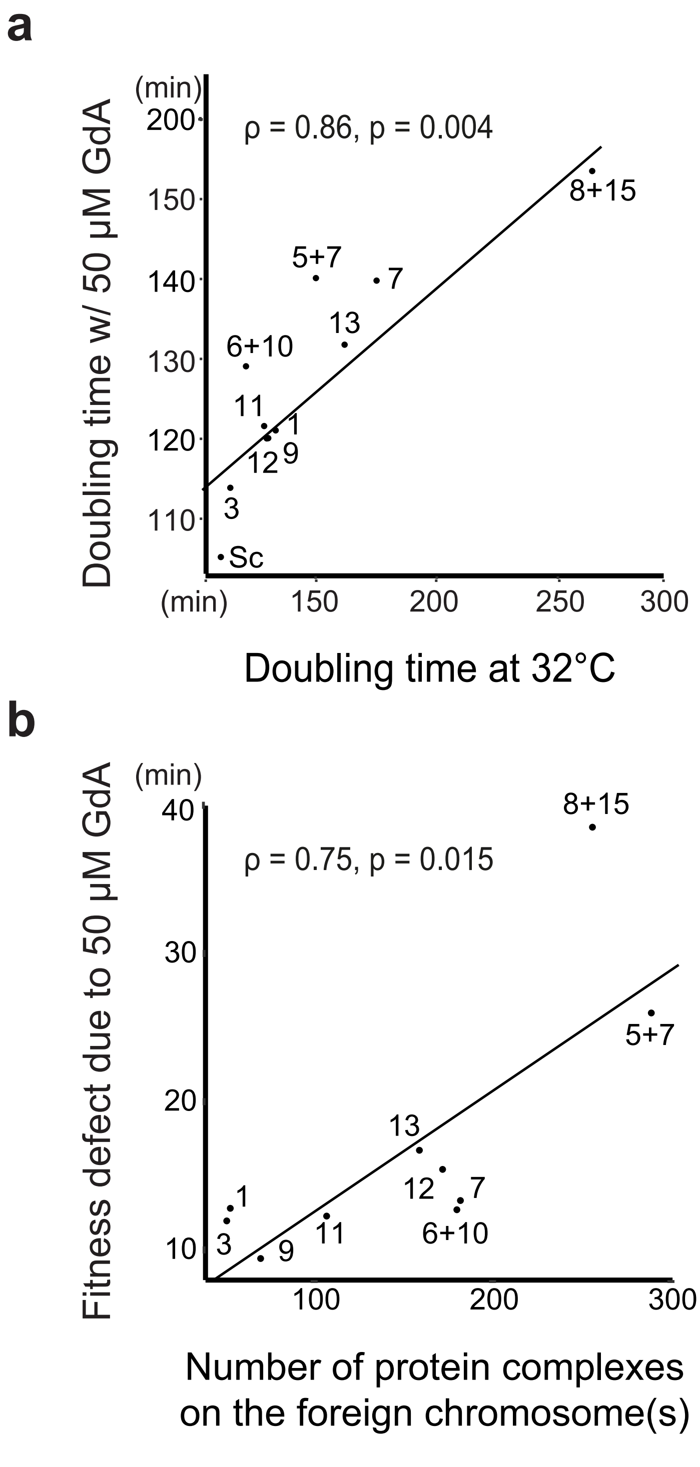

Supplementary Fig. 4: The intrinsic proteotoxic stress levels in replacement lines are correlated with the number of complexes having subunits encoded on the replaced chromosomes, even when the most defective line (16L) is excluded.

a, The fitness defect under treatment with Geldanamycin (50 µM GdA) is significantly correlated (Spearmans ρ = 0.86, p = 0.004) with the fitness defect due to mild heat stress (32°C) even when 16L is excluded (which exhibits the most severe growth defect among all replacement lines). **b,** The fitness defect under treatment with Geldanamycin (50 µM GdA) is significantly correlated (Spearmans ρ = 0.75, p = 0.015) with the number of complexes having subunits encoded on the replaced chromosomes, even without including data on 16L. Source data are provided as a Source Data file.

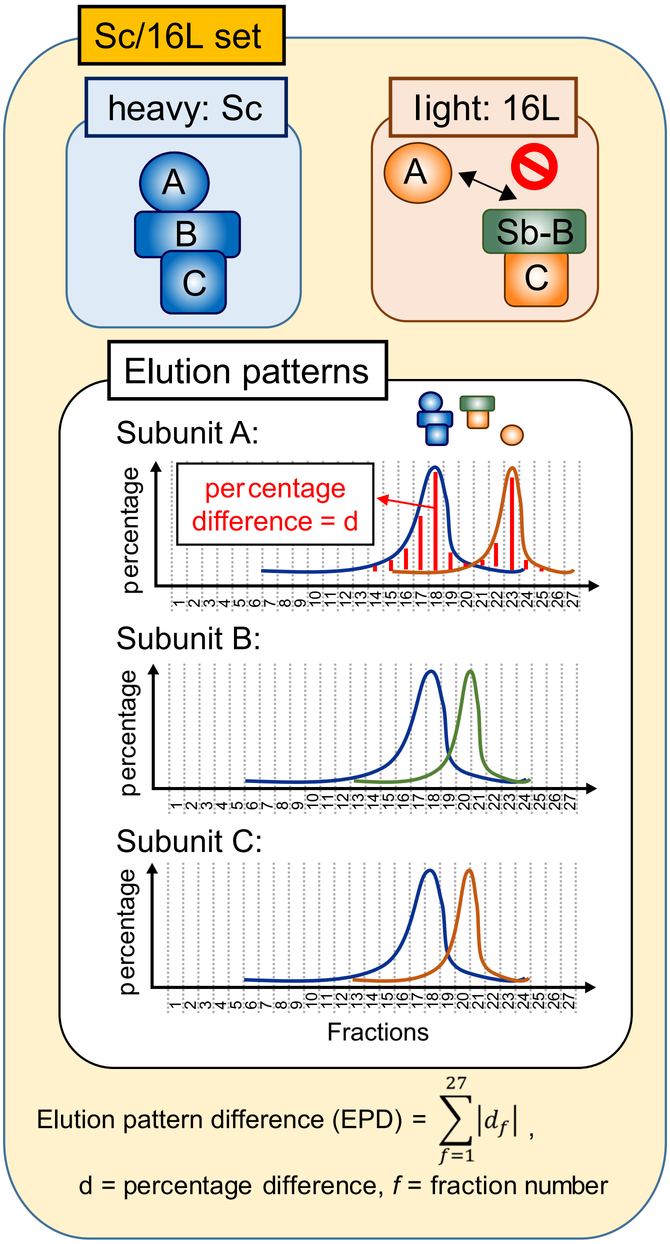

Supplementary Fig. 5: Illustrative representation of elution pattern differences (EPDs) using 16L as an example.

EPD represents the change of complex formation in 16L cells compared to the pure Sc strain. A hybrid protein complex containing subunits A, B, and C in 16L cells is shown as an example. Subunit B is encoded on Chromosome 16. Since Sb-B cannot interact properly with Sc-A in 16L cells, the chimeric complex is unstable, resulting in the dissociation of A from the BC subcomplex. After SEC, all components in the stable complex formed in Sc cells are eluted at a single peak (collections surrounding fraction 18), whereas the same proteins in 16L cells are eluted at different peaks of lower molecular weight or smaller sizes (collections surrounding fractions 20 and 23). By comparing these elution patterns, we can determine to what degree the complex has been disassembled. The differences are represented by the sum of the absolute value of the percentage difference in each fraction (d_f). The sum is denoted as the elution pattern difference (EPD) and ranges from 0 (completely overlapping peaks) to 2 (completely separate peaks).

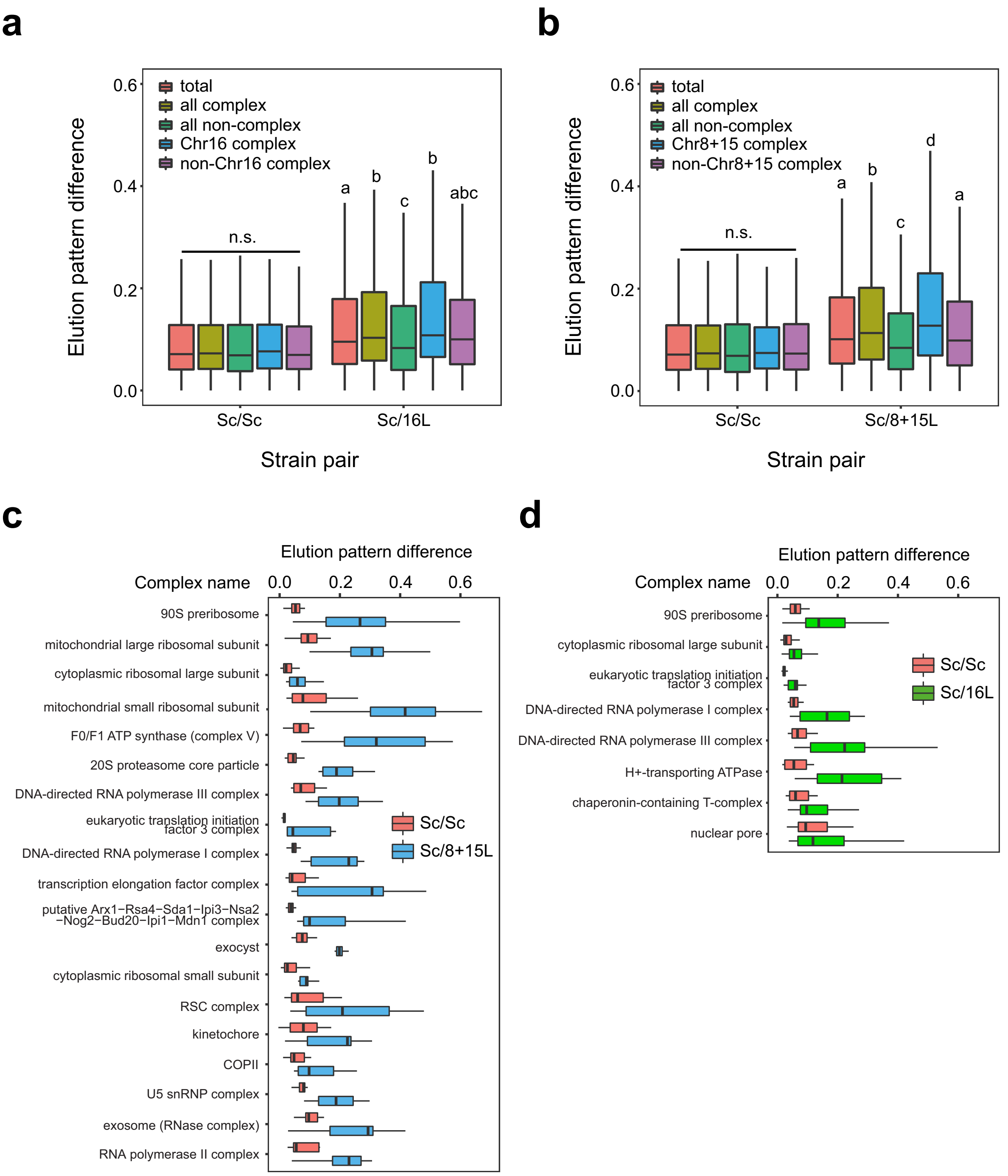

Supplementary Fig. 6: Similar destabilized protein complexes are observed in 8+15L and 16L cells even after excluding the proteins encoded by replaced chromosomes.
The protein complexes with subunits on Chromosomes 8+15 and Chromosome 16 are less stable than other complexes. To rule out the possibility that proteins encoded by Sb chromosomes might have different elution patterns contributing to observed EPDs in Fig. 3b and 3c, EPD values were recalculated after removing the proteins from Chromosome 16 **(a)** in both Sc-heavy/Sc-light (Sc/Sc) and Sc-heavy/16L-light (Sc/16L) sets or Chromosomes 8 and 15 **(b)** in both Sc-heavy/Sc-light (Sc/Sc) and Sc-heavy/8+15L-light (Sc/8+15L) sets. The distributions of EPD values for different groups of proteins of the Sc/Sc, Sc/8+15L and Sc/16L sets are shown in box plots. Distributions with the same letter (above each boxplot)

are not significantly different from each other (Dunn's pairwise tests with Bonferroni correction, p-values > 0.05, see Supplementary Data 11 for the p-values of all Dunn's pairwise tests). n.s.: not significant. **c** and **d**, After removing the proteins encoded by replaced chromosomes, 19 and 8 protein complexes are destabilized in 8+15L and 16L cells, respectively. The difference between these data and Fig. 3 (i.e., three complexes) is mainly due to decreased subunit numbers that reduce the statistical power in those complexes. Boxplots indicate median (middle line), 25th and 75th percentile (box), and min and max (whiskers). Statisitical information and raw data provided in Supplementary Data 6b and 12.

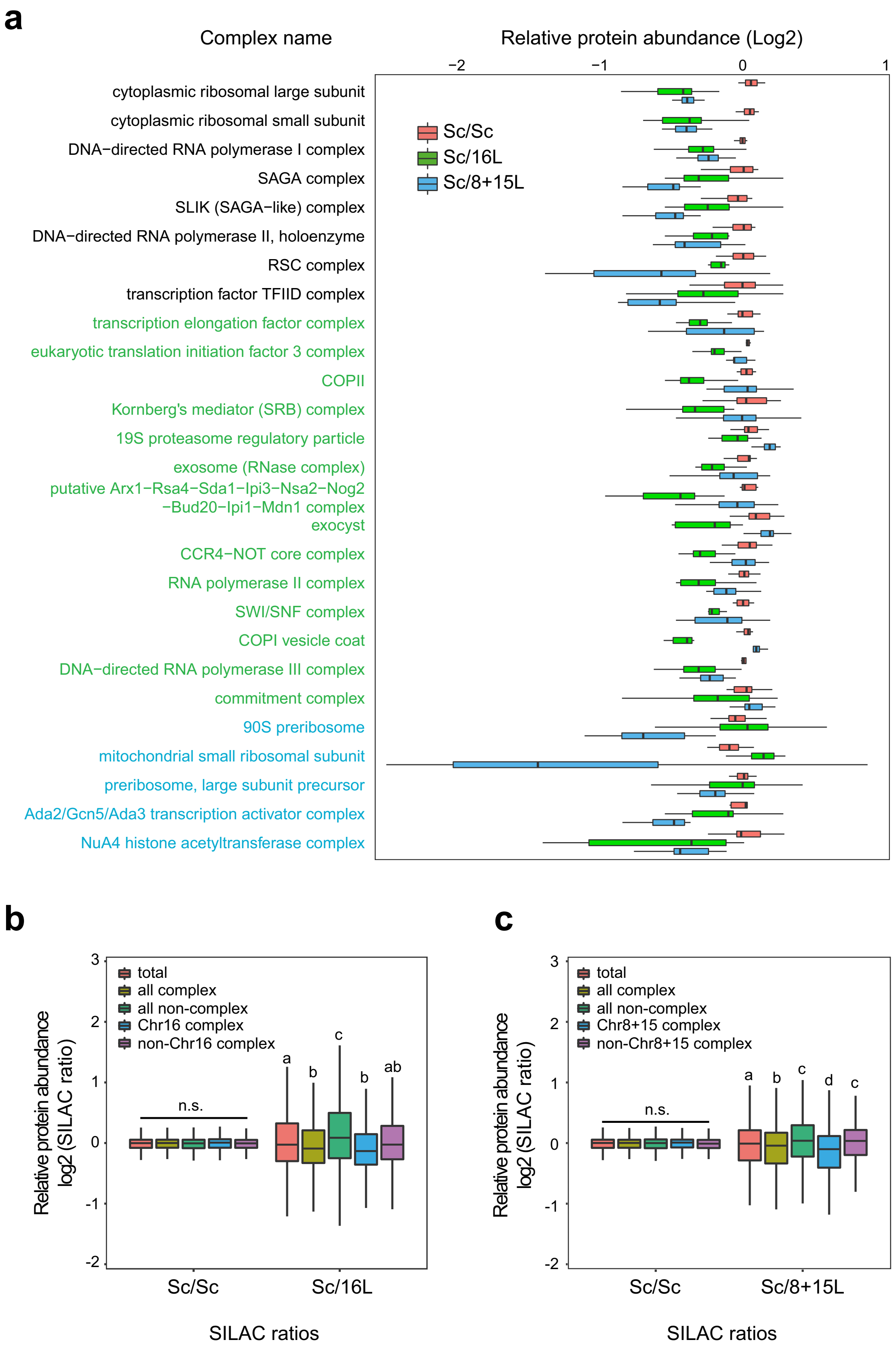

Supplementary Fig. 7: Protein abundances of complexes having subunits encoded on replaced chromosomes are significantly reduced even after excluding the proteins encoded by replaced chromosomes.

a, Twenty-seven protein complexes show significantly reduced subunit protein abundance in 8+15L and 16L cells. A protein complex was shown if the SILAC ratios of the complex subunits in the Sc/16L or Sc/8+15L sets were significantly lower than those in the Sc/Sc set. Shared, 16L-specific, and 8+15L-specific complexes are shown in back, green, and blue, respectively. **b** and **c,** To rule out the possible bias due to the Sb proteins, protein abundances for complex and non-complex proteins were compared after removing the proteins encoded on Chromosome 16 **(b)** or Chromosomes 8 and 15 **(c)**. Removing the proteins encoded by the replaced chromosomes did not change the patterns observed previously (Fig. 4a). Boxplots indicate median (middle line), 25th and 75th percentile (box), and min and max (whiskers). Statistical information and raw data are provided in Supplementary Data 11 and 12.

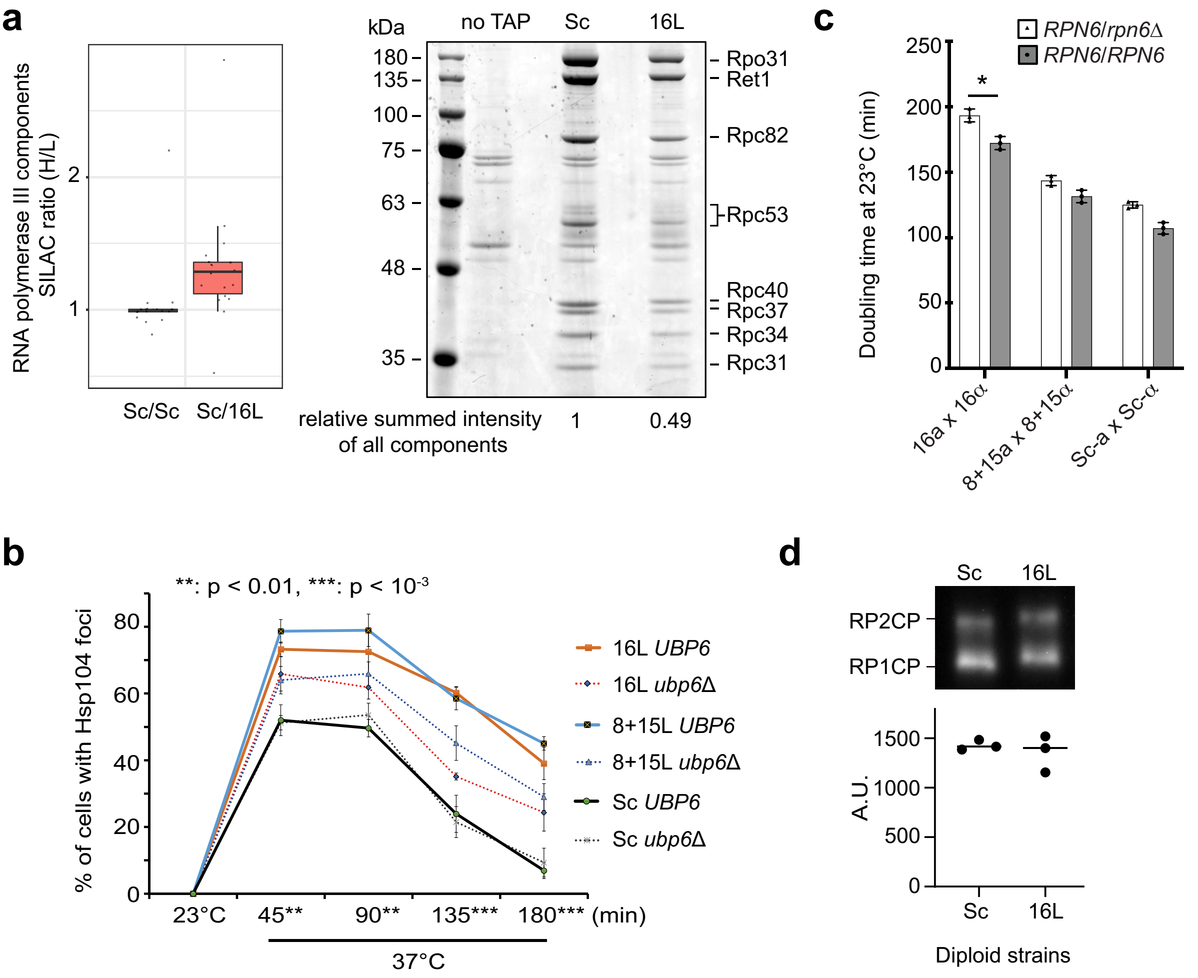

Supplementary Fig. 8: Assembled RNA Polymerase III is reduced and Proteasomes exhibit normal activity but are overburdened by destabilized complex components in replacement lines.

a, Formation of RNA polymerase III is impaired in 16L cells. The boxplot in the left panel depicts the SILAC ratios of protein components in RNA polymerase III from the Sc/16L and Sc/Sc sets. One of the RNA polymerase III subunits, Ret1, was tagged with TAP-tag, and the entire RNA polymerase III complex was purified from 16L and Sc cells and assessed by SDS-PAGE (right panel). An Sc strain without TAP-tagging was used as a control (NoTAP). The intensities of different bands were quantified using ImageJ. The ratio of the summed intensity of all components (Rpo31, Ret1, Rpc82, Rpc53, Rpc40, Rpc37, Rpc34, Rpc31) is 1:0.49. This complex purification experiment corroborates our proteomic data. **b,** The slow recovery of 16L and 8+15L diploid cells from heat stress is partially alleviated in *ubp6*Δ mutants. Yeast strains containing an *HSP104-mCherry-URA* cassette were grown to exponential phase in YPD at 23°C and then shifted to 37°C. The percentage of cells containing Hsp104-mCherry foci was determined at different time points (n = 8; N ≥ 500 cells per time point). **c,** The growth defect of 16L cells is aggravated in the heterozygous *RPN6/rpn6*Δ mutants at 23°C (n = 3). Rpn6 is an essential component of proteasomes, and the proteasomal degradation activity in *RPN6/rpn6*Δ mutants is mildly compromised. Cell lines were grown in YPD at 23°C and the doubling times were compared. **d,** The 26S proteasome holoenzymes from Sc and 16L cells were separated by the native gel and proteasome activities were assayed. Quantification of the total signal intensity from three

biological repeats is shown (n = 3). RP2CP: doubly-capped 26S proteasomes, RP1CP: singly-capped 26S proteasomes. A.U., absorbance units. The data are presented as mean values +/- SEM. *: p-value < 0.05, **: p-value < 0.01, ***: p-value < 10^{-3}, one-sided Student's t-test. Boxplots indicate median (middle line), 25th and 75th percentile (box), and min and max (whiskers). The summary of the boxplot in 8a is provided in Supplementary Data 12. Source data and detailed statistical information are provided as a Source Data file.

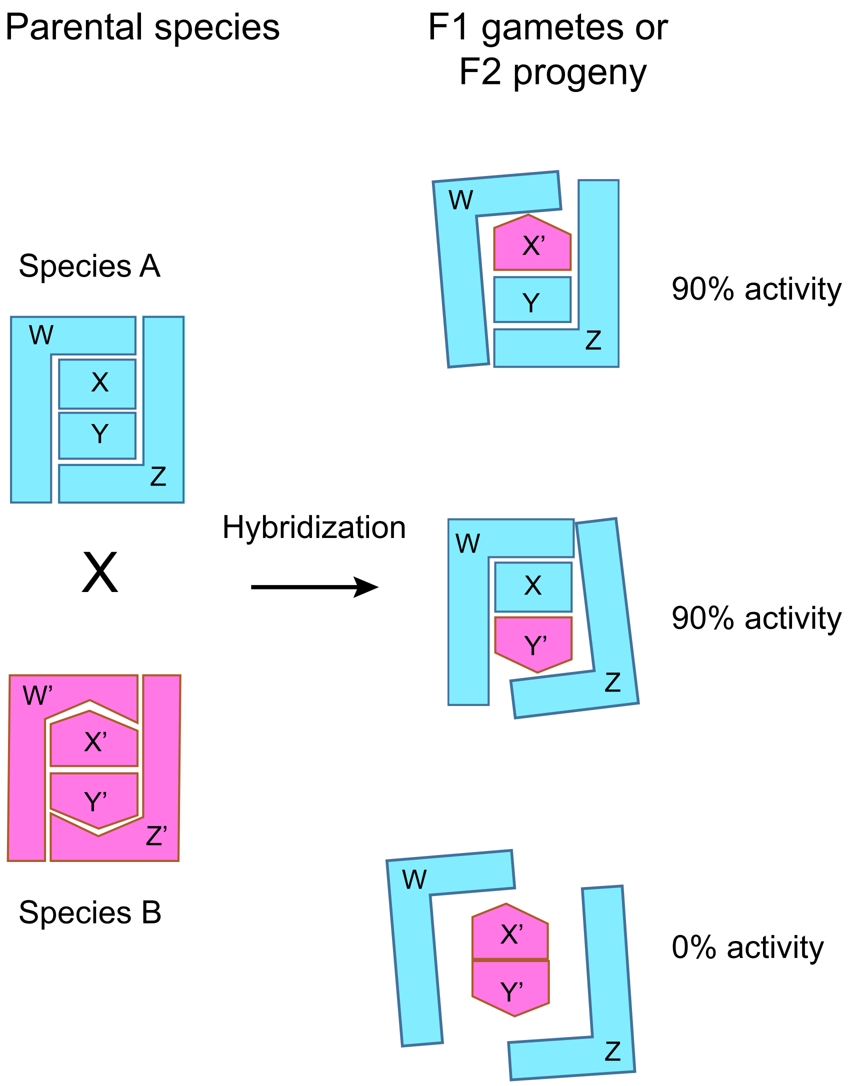

Supplementary Fig. 9: Protein complexes create a microenvironment for epistasis between subunits.

The structural stability of a complex is maintained by interactions between multiple subunits. As long as the chimeric complex is still assembled, it remains functional. However, the function of the chimeric complex may be completely lost if the chimeric complex contains multiple foreign subunits and its stability becomes compromised to an extent exceeding the buffering capacity.
