# Supplementary material for: Proteotoxicity caused by perturbed protein complexes underlies hybrid incompatibility in yeast
Source: Nat Commun. 2022 Jul 29;13:4394. doi: 10.1038/s41467-022-32107-4 (PMC9338014; doi:10.1038/s41467-022-32107-4)
Supplement: Supplementary file 3 — Description of Additional Supplementary Files [file 41467_2022_32107_MOESM3_ESM.pdf]

Description of Additional Supplementary Files

File Name: Supplementary Data 1
Description: Transcriptome data of replacement lines compared to the Sc strain. Log2-fold changes and the adjusted p-values are listed.

File Name: Supplementary Data 2
Description: Spearman's correlation between expression profiles of ESR genes from individual replacement lines, data on an aneuploid population from Tsai et al. (2019), and microarray results from Gasch et al. (2000).

File Name: Supplementary Data 3
Description: Gene Ontology of stable and unstable protein complex subunits.

File Name: Supplementary Data 4
Description: Protein complexes used in this study. The original dataset is from Costanzo et al. (2016). The modifications are provided in a separate sheet in the file.

File Name: Supplementary Data 5
Description: Proteome data from the SILAC-SEC-MS experiment along with the EPD value for each protein.

File Name: Supplementary Data 6
Description: List of protein complexes tested in this study.

File Name: Supplementary Data 7
Description: List of protein complexes with reduced subunit protein abundance.

File Name: Supplementary Data 8
Description: Spearman's correlation between transcriptomes and proteomes of 8+15L and 16L.

File Name: Supplementary Data 9
Description: List of primers used in this study.

File Name: Supplementary Data 10
Description: List of strains used in this study.

File Name: Supplementary Data 11
Description: P-values of all Dunn's pairwise tests of proteomics data.

File Name: Supplementary Data 12
Description: Data used to plot the boxplots in Figures 3, 4, and Supplementary Figures 6, 7, and 8.
