# Supplementary material for: Proteotoxicity caused by perturbed protein complexes underlies hybrid incompatibility in yeast
Source: Nat Commun. 2022 Jul 29;13:4394. doi: 10.1038/s41467-022-32107-4 (PMC9338014; doi:10.1038/s41467-022-32107-4)
Supplement: Supplementary file 5 — Reporting Summary [file 41467_2022_32107_MOESM5_ESM.pdf]

nature portfolio

Corresponding author(s): Jun-Yi Leu

Last updated by author(s): 2022/07/03

Reporting Summary

Nature Portfolio wishes to improve the reproducibility of the work that we publish. This form provides structure for consistency and transparency in reporting. For further information on Nature Portfolio policies, see our Editorial Policies and the Editorial Policy Checklist.

Statistics

For all statistical analyses, confirm that the following items are present in the figure legend, table legend, main text, or Methods section.

n/a	Confirmed	
☐	☒	The exact sample size (*n*) for each experimental group/condition, given as a discrete number and unit of measurement
☐	☒	A statement on whether measurements were taken from distinct samples or whether the same sample was measured repeatedly
☐	☒	The statistical test(s) used AND whether they are one- or two-sided<br>*Only common tests should be described solely by name; describe more complex techniques in the Methods section.*
☒	☐	A description of all covariates tested
☐	☒	A description of any assumptions or corrections, such as tests of normality and adjustment for multiple comparisons
☐	☒	A full description of the statistical parameters including central tendency (e.g. means) or other basic estimates (e.g. regression coefficient) AND variation (e.g. standard deviation) or associated estimates of uncertainty (e.g. confidence intervals)
☐	☒	For null hypothesis testing, the test statistic (e.g. *F*, *t*, *r*) with confidence intervals, effect sizes, degrees of freedom and *P* value noted<br>*Give P values as exact values whenever suitable.*
☒	☐	For Bayesian analysis, information on the choice of priors and Markov chain Monte Carlo settings
☒	☐	For hierarchical and complex designs, identification of the appropriate level for tests and full reporting of outcomes
☒	☐	Estimates of effect sizes (e.g. Cohen's *d*, Pearson's *r*), indicating how they were calculated

Our web collection on statistics for biologists contains articles on many of the points above.

Software and code

Policy information about availability of computer code

Data collection	Collection of sequencing was performed using the Illumina platform
Data analysis	All the softwares used to analyze data in this work are open source.<br>Details of the opensource softwares used in this study are listed in the manuscript.<br>No custom made code was developed for analysis and this is also stated in the manuscript.<br>Basic program scripts in R (version 3.3), Perl or Python for parsing and plotting.<br>Growth assays: Tecan, Mannedorf, Switzerland<br>RNA Sequencing data analysis: Trimmomatic (version 0.38), Salmon (version 0.12.0) and DESeq2 (version 1.22.2).<br>Proteomics data analysis: MaxQuant software (version 1.6.7.0).<br>ImageJ: for microscopy data analysis.

For manuscripts utilizing custom algorithms or software that are central to the research but not yet described in published literature, software must be made available to editors and reviewers. We strongly encourage code deposition in a community repository (e.g. GitHub). See the Nature Portfolio guidelines for submitting code & software for further information.

Data

Policy information about availability of data

All manuscripts must include a data availability statement. This statement should provide the following information, where applicable:

- Accession codes, unique identifiers, or web links for publicly available datasets
- A description of any restrictions on data availability
- For clinical datasets or third party data, please ensure that the statement adheres to our policy

Proteomic data are available via ProteomeXchange with identifier PXD028358 (http://www.ebi.ac.uk/pride/archive/projects/PXD028358).
RNA-seq data are available via BioProject PRJNA855266 (https://www.ncbi.nlm.nih.gov/bioproject/?term=PRJNA855266). We have now uploaded the data and cited the accession number in the Data availability section of the manuscript. Also, All the datasets used in our study are provided as Supplementary Data and Source Data files.

Field-specific reporting

Please select the one below that is the best fit for your research. If you are not sure, read the appropriate sections before making your selection.

☒ Life sciences ☐ Behavioural & social sciences ☐ Ecological, evolutionary & environmental sciences

For a reference copy of the document with all sections, see nature.com/documents/nr-reporting-summary-flat.pdf

Life sciences study design

All studies must disclose on these points even when the disclosure is negative.

Sample size	Sample size was chosen based on previously published literature on the same subject. Three or more to enable calculation of standard deviation and significance. Four replicates was used for RNA Sequencing. Three or more repeats were used for all the experiments. Exception to this was for LC MS/MS experiments, where proteins in the 27 fractions from size exclusion chromatography were used.<br>Minimum Sample-size (n) used in this study is 3. For several experiments n > 3. Experiments were done using three repeats to account for standard deviation and variance in the data and also to assess the significance of the statistical test used.<br>We have now indicated the exact sample size from each experiments in the legends and also provided the all the data used in this study in the Source Data file.
Data exclusions	No data was excluded.
Replication	Reproducibility was confirmed. Each experiment was repeated at least three times and the data was consistent and reproducible. We have also added the Statistics and Reproducibility section at the end of Methods section and have also added reproducibility in appropriate legends. All the data sets used for analysis and making of the figures are now provided in the Source Data file.
Randomization	Samples were not randomized, appropriate controls are included in each figure. Hypothesis testing of experimental data is mainly performed using one-sided students T-test and Spearman's correlation coefficient. Here the test was performed each time against their respective appropriate control variables.<br>For RNA-seq data analysis all the samples and the controls were normalized independently and these independently normalized TPM values were used for generating fold change and test for significant deviation in their expression levels. Furthermore the variation between the repeats were accounted for in the through Bayesian inference method in built in DESeq2 (version 1.22.2) tool used for expression analysis.
Blinding	Blinding is not relevant to this study, which does not include any patient or clinical assessments. Otherwise, for image quantifications, the analysis was performed blindly. For data collection of image quantification, investigators were blinded to group allocation and during data collection analysis.

Reporting for specific materials, systems and methods

We require information from authors about some types of materials, experimental systems and methods used in many studies. Here, indicate whether each material, system or method listed is relevant to your study. If you are not sure if a list item applies to your research, read the appropriate section before selecting a response.

Materials & experimental systems

n/a	Involved in the study
☒	☐ Antibodies
☒	☐ Eukaryotic cell lines
☒	☐ Palaeontology and archaeology
☐	☒ Animals and other organisms
☒	☐ Human research participants
☒	☐ Clinical data
☒	☐ Dual use research of concern

Methods

n/a	Involved in the study
☒	☐ ChIP-seq
☒	☐ Flow cytometry
☒	☐ MRI-based neuroimaging

Animals and other organisms

Policy information about studies involving animals; ARRIVE guidelines recommended for reporting animal research

Laboratory animals	Yeast Saccharomyces cerevisiae (W303 strain) and Saccharomyces var uvarum (S. bayanus #180) collected by Dr. Duccio Cavalieri, University of Florence, Italy) and hybrids of the above strains.
Wild animals	None
Field-collected samples	None
Ethics oversight	No ethical guidance or approval was required as yeasts used in this study are standard laboratory model organisms.

Note that full information on the approval of the study protocol must also be provided in the manuscript.
